# Inhibitor clinical burden of disease: a comparative analysis of the CHESS data

**DOI:** 10.1186/s13023-018-0929-9

**Published:** 2018-11-09

**Authors:** Abiola O. Oladapo, Mei Lu, Shaun Walsh, Jamie O’Hara, Teresa L. Kauf

**Affiliations:** 1 Outcomes Research & Epidemiology, Shire, 650 E Kendall Street, Cambridge, MA 02142 USA; 2 HCD Economics, Daresbury, UK; 3 Faculty of Health and Social Care, University of Chester, Chester, UK; 4 Shire, Zug, Switzerland

**Keywords:** Hemophilia, Inhibitors, Disease burden

## Abstract

**Background:**

Patients with hemophilia and inhibitors generally face greater disease burden compared to patients without inhibitors. While raising awareness of relative burden may improve the standard of care for patients with inhibitors, comparative data are sparse. Analyzing data drawn from the Cost of Haemophilia across Europe – a Socioeconomic Survey (CHESS) study, the aim of this study was to compare the clinical burden of disease in patients with severe hemophilia with and without inhibitors. Hemophilia specialists (*N* = 139) across five European countries completed an online survey between January–April 2015, providing demographic, clinical and 12-month ambulatory/secondary care activity data for 1285 patients. Patients with hemophilia who currently presented with inhibitors and those who never had inhibitors were matched on baseline characteristics via propensity score matching. Outcomes were compared between the two cohorts using a paired t-test or Wilcoxon signed-rank or McNemar’s test.

**Results:**

The proportion of patients who currently presented with inhibitors was 4.5% (58/1285). Compared to PS-matched patients without inhibitors, patients with inhibitors experienced more than twice the mean annual number of bleeds (mean ± standard deviation, 8.29 ± 9.18 vs 3.72 ± 3.95; *p* < .0001) and joint bleeds (2.17 ± 1.90 vs 0.98 ± 1.15; *p* < .0001), and required more hemophilia-related (mean ± standard deviation, 1.79 ± 1.83 vs 0.64 ± 1.13) and bleed-related hospitalizations (1.86 ± 1.88 vs 0.81 ± 1.26), hemophilia-related consultations (9.30 ± 4.99 vs 6.77 ± 4.47), and outpatient visits (22.09 ± 17.77 vs 11.48 ± 16.00) (all, *p* < .001). More than one-half (53.5%) experienced moderate/severe pain necessitating medication compared to one-third (32.8%) of patients without inhibitors (*p* = .01).

**Conclusions:**

Patients with hemophilia and inhibitors exhibited greater clinical burden and higher resource utilization compared to their peers without inhibitors. Strategies for improving the standard of care may alleviate burden in this population.

**Electronic supplementary material:**

The online version of this article (10.1186/s13023-018-0929-9) contains supplementary material, which is available to authorized users.

## Background

Congenital hemophilia is a life-long, X-linked hereditary bleeding disorder caused by the deficiency of coagulation factor VIII (FVIII) (hemophilia A) or factor IX (FIX) (hemophilia B) [1]. More than 400,000 individuals are afflicted globally [2] among whom, depending on the population, approximately 33% to 50% have severe hemophilia (FVIII or FIX activity level < 1% of normal) [3]. Severe hemophilia typically manifests during childhood or adolescence, peak periods of growth and psychosocial development, and is clinically characterized by a heightened risk for recurrent, spontaneous bleeding into the joints and muscles [1, 4]. Without adequate management, patients can suffer significant morbidity due to the development of chronic arthropathy, disability, and impaired health-related quality of life (HRQoL). Additionally, patients with severe hemophilia can sustain substantial societal losses owing to decreased school/work participation, diminished productivity, and increased caregiver burden [5–7]. The Social Economic Burden and Health-Related Quality of Life of Patients with Rare Diseases (BURQOL-RD) study recently examined a cross-section of patients with hemophilia (*N* = 339) and caregivers (*N* = 62) in Europe and found that 40% of patients reported some degree of physical disability (Barthel Index score ≥ 91), and over half (58.9%) of caregivers felt burdened [8].

Advancements in hemophilia care have yielded factor replacement therapies, such as recombinant anti-hemophilia FVIII and recombinant FIX, which when administered prophylactically are efficacious in preventing or reducing the risk of bleeding and serious bleeding complications, preserving joint function, and improving HRQoL and productivity in patients with severe hemophilia [9–14]. Following exposure to exogenous FVIII/FIX, however, approximately 10–30% of patients with severe hemophilia A and 2–5% of those with severe hemophilia B develop alloantibodies to FVIII and FIX, respectively [15]. These inhibitors neutralize the coagulant activity of infused FVIII or FIX, rendering patients refractory to standard factor replacement therapy.

The development of a high-titer inhibitor (> 5 Bethesda units) in particular poses significant treatment challenges as achieving hemostasis becomes difficult with the administration of FVIII or FIX concentrates, necessitating the use of bypassing agents to control and prevent bleeding episodes. Current clinical guidance supports treatment with bypassing agents prophylaxis (BAP) in patients with severe hemophilia and inhibitors given its benefits over on-demand treatment [16]. Compared to on-demand treatment only, BAP reduces the frequency of joint bleeds, prevents the development of new target joints, reduces hospital admissions and school/work absences, and improves HRQoL [17–22]. However, despite the benefits of prophylaxis, and contrary to standard of care for patients without inhibitors, the majority of patients with inhibitors are still managed on-demand [23–25]. While it is generally accepted that patients with inhibitors face a higher disease burden compared to patients without inhibitors, limited comparative data exist in the literature to further raise awareness of the crucial need to improve the standard of care for these patients relative to their peers without inhibitors [26].

In this study, we used patient-level data from the Cost of Haemophilia across Europe – a Socioeconomic Survey (CHESS) study [27] to compare bleed rates and health resource utilization (HRU) in patients with severe hemophilia with and without inhibitors. As frequent evaluation of disease burden may assist in prioritizing and improving patient care [23], the objective was to quantify the real-world clinical burden of inhibitors in this contemporary population with severe hemophilia.

## Methods

### Data collection

The CHESS study was conducted by the University of Chester in partnership with the Haemophilia Society (United Kingdom). The CHESS Steering Committee, a collaboration of treating clinicians, patients with hemophilia, and representatives from academia and hemophilia societies, provided governance and oversight to ensure high standards of quality. As such, the CHESS study represents the first comprehensive, ‘bottom-up’ cost-of-illness study that captured data from approximately 15% of patients with severe hemophilia across five European countries (EU5) – France, Germany, Italy, Spain, and the United Kingdom. Its aim was to quantify the real-world societal costs of severe hemophilia (factor level of < 1%) in the EU5 as a basis for understanding the potential impact of new hemophilia treatments.

Between January and April 2015, a cross-section of 139 hemophilia specialists completed an on-line survey and provided demographic, clinical, and 12-month retrospective ambulatory and secondary care activity data for adult males (> 18 years) with severe hemophilia A (*N* = 996) or B (*N* = 289). Of the 1285 patients, 551 filled out corresponding questionnaires and disclosed information on out-of-pocket expenses and HRU. All patient-level data were anonymized. The current analysis was restricted to the patients and outcomes specified herein. Outcome variables for the analysis were defined a priori and were determined based on input from expert hematologists and the hemophilia literature.

#### Identification of patients with and without inhibitors

The present analysis included the CHESS patients who currently presented with inhibitors and those who had never had an inhibitor. Patients with inhibitors were identified from affirmative physician responses to the query, “Is the patient currently diagnosed with an inhibitor?” For patients without inhibitors, physicians responded ‘Never’ to the question, “How many times has the patient developed an inhibitor to factor replacement therapies over their lifetime?”

### Physician-reported information

Physicians provided data on patient demographics and clinical characteristics (hemophilia type and comorbidities), 12-month bleed rates (including major, minor, and joint bleeds), and the current status of the patient’s hemophilia-related chronic pain. Comorbidities of interest were selected by the CHESS Steering Committee. Minor bleeds were defined as bleeds resolving within 24 h of treatment and associated with mild pain, minimal swelling and restriction of motion. Bleeds failing to respond to treatment within 24 h and causing pain, effusion, and limitation of motion were considered major bleeds. The patient’s current pain level was described as: 1) No pain: no functional deficit, no analgesic use (except with acute hemarthrosis); 2) Mild pain: does not interfere with occupation nor with activities of daily living (ADL), may require occasional non-narcotic analgesic; 3) Moderate pain: partial or occasional interference with occupation or ADL, uses non-narcotic medications; or 4) Severe pain: interferes with occupation or ADL, requires frequent use of non-narcotic and narcotic medications. Physicians indicated their current satisfaction with the patient’s prognosis by selecting 1 of 3 responses: 1) Satisfied; 2) Not satisfied, but I believe this is the best that can be realistically achieved for this patient; or 3) Not satisfied, and I believe better outcomes can be achieved for this patient.

### Health resource utilization

Physicians reported the patient’s frequency of HRU over the past 12 months, including hemophilia-related scheduled and non-scheduled consultations, hemophilia- and non-hemophilia-related outpatient visits, and hemophilia- and bleed-related hospitalizations. ‘Hemophilia-related’ services pertained to treatment for hemophilia complications or acute events, or planned surgeries.

### Statistical analysis

Statistical analyses were performed using SAS 9.3 (SAS Institute Inc., Cary, NC, US). Baseline patient demographics, clinical attributes, and outcome measures were summarized descriptively as the mean ± standard deviation (SD) and median (range) for continuous variables and frequency (percentage) for categorical variables. The two study cohorts (i.e. inhibitors and non-inhibitors) were matched using propensity score (PS) matching. In the PS matching, a logistic regression model was used to predict the odds for each patient to be enrolled in the inhibitor cohort, given patient characteristics (e.g. age, body mass index, comorbidity). Age and body mass index were treated as independent, continuous variables while race was dichotomized as either white or non-white. Each comorbidity variable was introduced into the model as a dichotomous variable. A greedy propensity score matching approach, utilizing the smaller caliper width that maintained the maximum sample size (caliper size of 0.035), was used to match a patient in the inhibitor cohort to a patient in the non-inhibitor cohort who had the closest propensity score within the specified caliper size and having the same type of hemophilia (hemophilia A or B). To determine if balance was achieved between the matched groups, differences between the matched pairs were evaluated for each baseline variable using a paired t-test or signed-rank test for continuous data and the McNemar’s test for binary data. Finally, to address the study objective, matched cohorts were then compared on each outcome variable using a paired t-test or the Wilcoxon signed-rank test for continuous variables and the McNemar’s test or exact McNemar’s test for categorical variables. A two-tailed *p*-value < 0.05 was considered statistically significant.

## Results

### Baseline demographics and clinical characteristics

Fifty-eight (4.5%) of the 1285 CHESS patients currently had an inhibitor, and 1091 (84.9%) had never developed an inhibitor (Table 1). The remaining patients (10.6%) used to but no longer have inhibitors. Among the 1149 patients (i.e. those that currently have or never had inhibitors), the mean age was 35.5 ± 14.82 years; 87.6% were white. Overall, 61.8% of patients were employed full-time; 3.0% were either unable to work or were currently on a temporary leave of absence due to their hemophilia. Nearly half (49%) of the patients had at least one comorbidity of which, anxiety was most common (14.1%), followed by depression (13.1%), and hypertension (12.1%).

**Table 1:** Baseline characteristics of CHESS sample with severe hemophilia and unmatched cohorts with and without inhibitors

Characteristic	All patients	Patients who never developed inhibitors	Patients with current inhibitors	*p*-value^a^
	*N* = 1149	*N* = 1091	*N* = 58	
Demographics				
Age, years				
Mean ± SD	35.50 ± 14.82	35.16 ± 14.74	41.90 ± 14.95	.0002
Median (range)	32 (18.00–88.00)	31 (18.00–88.00)	39.5 (18.00–80.00)	
Race, *N* (%)				
White	1007 (87.6)	958 (87.8)	49 (84.5)	.6260
African	50 (4.4)	48 (4.4)	2 (3.4)	
Asian-Indian subcontinent	37 (3.2)	34 (3.1)	3 (5.2)	
Asian-Other	6 (0.5)	6 (0.5)	0 (0.0)	
Middle Eastern	46 (4.0)	42 (3.8)	4 (6.9)	
Other	3 (0.3)	3 (0.3)	0 (0.0)	
White	1007 (87.6)	958 (87.8)	49 (84.5)	.4532
Non-white	142 (12.4)	133 (12.2)	9 (15.5)	
Body mass index				
Mean ± SD	24.76 ± 3.25	24.73 ± 3.27	25.31 ± 2.78	.1140
Median (range)	24.57 (14.11–57.47)	24.51 (14.11–57.47)	25 (20.52–32.83)	
Education, *N* (%)				
None	15 (1.3)	12 (1.1)	3 (5.2)	
Primary	51 (4.4)	49 (4.5)	2 (3.4)	
Secondary	8 (0.7)	8 (0.7)	0 (0.0)	
Undergraduate	76 (6.6)	73 (6.7)	3 (5.2)	
Graduate	203 (17.7)	192 (17.6)	11 (19.0)	
Did not answer	129 (11.2)	119 (10.9)	10 (17.2)	
College or graduate				
Yes	180 (38.5)	168 (38.1)	12 (46.2)	<.0001
No	287 (61.5)	273 (61.9)	14 (53.8)	
Employment status, *N* (%)				
Full-time employed	182 (15.8)	174 (15.9)	8 (13.8)	
Homemaker	1 (0.1)	1 (0.1)	0 (0.0)	
Self-employed	3 (0.3)	1 (0.1)	2 (3.4)	
Other: Not determined/not specified	4 (0.4)	4 (0.4)	0 (0.0)	
Part-time employed	74 (6.4)	68 (6.2)	6 (10.3)	
Retired	46 (4.0)	42 (3.8)	4 (6.9)	
Student	84 (7.3)	82 (7.5)	2 (3.4)	
Temporary leave of absence due to my hemophilia	6 (0.5)	5 (0.5)	1 (1.7)	
Temporary leave of absence due to other reason(s)	6 (0.5)	6 (0.5)	0 (0.0)	
Unable to work due to my hemophilia	29 (2.5)	23 (2.1)	6 (10.3)	
Unable to work due to other reason(s)	2 (0.2)	2 (0.2)	0 (0.0)	
Unemployed, able to work	40 (3.5)	40 (3.7)	0 (0.0)	
Full-time employed				
Yes	182 (38.2)	174 (38.8)	8 (28.6)	<.0001
No	295 (61.8)	275 (61.2)	20 (71.4)	
Hemophilia type, *N* (%)				
Hemophilia A	894 (77.8)	847 (77.6)	47 (81.0)	.5438
Hemophilia B	255 (22.2)	244 (22.4)	11 (19.0)	
Comorbidities, *N* (%)				
None	586 (51.0)	578 (53.0)	8 (13.8)	<.0001
Alcohol dependence	39 (3.4)	35 (3.2)	4 (6.9)	.1291
Anemia	65 (5.7)	57 (5.2)	8 (13.8)	.0135
Anxiety	162 (14.1)	150 (13.7)	12 (20.7)	.1389
Depression	151 (13.1)	140 (12.8)	11 (19.0)	.1779
Diabetes mellitus	60 (5.2)	52 (4.8)	8 (13.8)	.0084
Fibromyalgia	41 (3.6)	34 (3.1)	7 (12.1)	.0034
Hepatitis B virus	20 (1.7)	19 (1.7)	1 (1.7)	1.0000
Hepatitis C virus	61 (5.3)	56 (5.1)	5 (8.6)	.2282
Human immunodeficiency virus	31 (2.7)	28 (2.6)	3 (5.2)	.2028
Hypertension	139 (12.1)	126 (11.5)	13 (22.4)	.0134
Hypercholesterolemia	70 (6.1)	65 (6.0)	5 (8.6)	.3931
Ischemic heart disease	19 (1.7)	16 (1.5)	3 (5.2)	.0665
Obesity	63 (5.5)	60 (5.5)	3 (5.2)	1.0000
Osteoarthritis	68 (5.9)	60 (5.5)	8 (13.8)	.0175
Osteoporosis	11 (1.0)	11 (1.0)	0 (0.0)	1.0000
Rheumatoid arthritis	9 (0.8)	9 (0.8)	0 (0.0)	1.0000
Other	18 (1.6)	16 (1.5)	2 (3.4)	.2293

*Abbreviations*: *CHESS*, Cost of Haemophilia across Europe – a Socioeconomic Survey; *SD*, standard deviation

^a^*P*-values were derived from a paired t-test or Wilcoxon signed rank test for continuous post-match variables and the McNemar’s test or exact McNemar’s test for categorical variables; *p* < .05 indicates statistical significance

In the unmatched cohorts, patients with inhibitors were older (mean age, 41.90 ± 14.95 years vs 35.16 ± 14.74 years; *p* < .0002) and had significantly higher frequencies of anemia (*p* = .0135), diabetes mellitus (*p* = .0084), fibromyalgia (*p* = .0034), hypertension (*p* = .0134), and osteoarthritis (*p* = .0175). Among the patients with relevant data, a significantly greater proportion of patients with inhibitors had attended college/graduate school (46.2% [12/26] vs 38.1% [168/441], respectively; *p* < .0001); however, fewer patients with inhibitors worked full-time (28.6% [8/28] vs 38.8% [174/449]; *p* < .0001). Following PS matching (Table 2), the demographic and clinical attributes appeared statistically balanced between the cohorts (each, *N* = 58).

**Table 2:** Baseline characteristics of PS-matched patients with and without inhibitors in the CHESS study^a^

Characteristic	Patients who never developed inhibitors	Patients with current inhibitors	*p*-value^b^
	*N* = 58	*N* = 58	
Demographics			
Age, years			
Mean ± SD	43.71 ± 17.17	41.90 ± 14.95	.4800
Median (range)	43 (18.00–88.00)	39.5 (18.00–80.00)	
Race, *N* (%)			
White	43 (74.1)	49 (84.5)	.1573
Body mass index			
Mean ± SD	24.92 ± 2.67	25.31 ± 2.78	.4072
Median (range)	25.08 (15.57–32.02)	25.00 (20.52–32.83)	
Hemophilia type, *N* (%)			
Hemophilia A	47 (81.0)	47 (81.0)	1.0000
Comorbidities, *N* (%)			
Alcohol dependence	7 (12.1)	4 (6.9)	.3173
Anemia	6 (10.3)	8 (13.8)	.5271
Anxiety	13 (22.4)	12 (20.7)	.8415
Depression	13 (22.4)	11 (19.0)	.6374
Diabetes mellitus	7 (12.1)	8 (13.8)	.7389
Fibromyalgia	3 (5.2)	7 (12.1)	.1573
Hepatitis B virus	1 (1.7)	1 (1.7)	1.0000
Hepatitis C virus	9 (15.5)	5 (8.6)	.2850
Human immunodeficiency virus	8 (13.8)	3 (5.2)	.0956
Hypertension	15 (25.9)	13 (22.4)	.6374
Hypercholesterolemia	7 (12.1)	5 (8.6)	.5271
Ischemic heart disease	4 (6.9)	3 (5.2)	.7055
Obesity	1 (1.7)	3 (5.2)	.3173
Osteoarthritis	7 (12.1)	8 (13.8)	.7815

*Abbreviations*: *BMI* body mass index, *CHESS* Cost of Haemophilia across Europe – a Socioeconomic Survey, *PS* propensity score, *SD* standard deviation

^a^Patients with current inhibitors were matched to patients who had never developed an inhibitor based on demographics (age, BMI, race) and comorbidity status using propensity scores stratified by hemophilia type. Matching was performed using a preset caliper size of 0.035 to maintain the maximum sample size using the smallest caliper width

^b^*P*-values were derived from a paired t-test or Wilcoxon signed rank test for continuous post-match variables and the McNemar’s test or exact McNemar’s test for categorical variables; *p* < .05 indicates statistical significance

### Bleeding outcomes following PS-matching

**Fig. 1:**
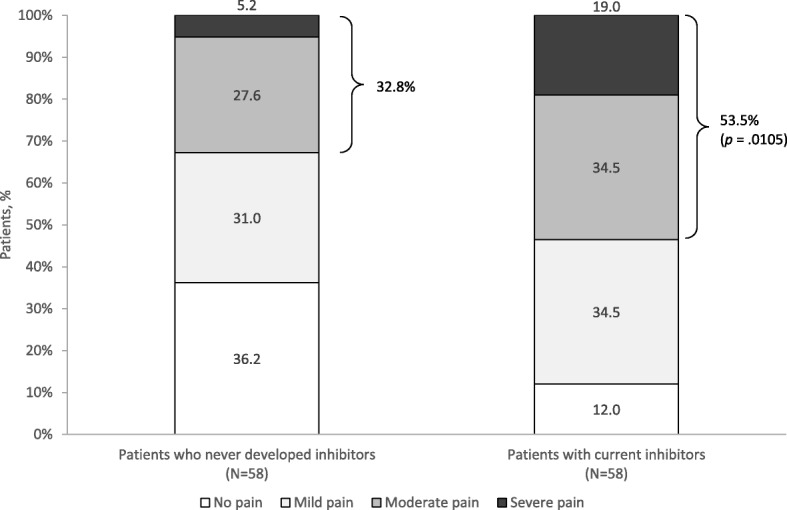
Physician-reported chronic hemophilia-pain by severity for PS-matched patients with and without inhibitors in CHESS study^a^. ^a^Patients with current inhibitors were matched to patients who had never developed an inhibitor based on demographics (age, BMI, race) and comorbidity status using propensity scores stratified by hemophilia type. Matching was performed using a preset caliper size of 0.035 to maintain the maximum sample size using the smallest caliper width. ^b^*P*-value refers to the difference in the proportion of patients with moderate to severe pain between cohorts and was derived from the McNemar’s test for categorical variables; *p* < .05 indicates statistical significance. Abbreviations: BMI, body mass index; CHESS, Cost of Haemophilia across Europe – a Socioeconomic Survey; PS, propensity score

In the PS-matched analysis (Table 3), the mean annualized bleed rate (ABR) in patients with inhibitors was more than doubled that in patients without inhibitors (8.29 ± 9.18 vs 3.72 ± 3.95; *p* < .0001); 81% of patients with inhibitors had experienced a major bleed (vs 37.9% of patients without inhibitors; *p* < .0001), and all patients with inhibitors (100%) had minor bleeds (vs 82.8% of patients without inhibitors; *p* = .0047). Similarly, the mean annualized joint bleed rate (AJBR) in patients with inhibitors exceeded that in patients without inhibitors by more than two-fold (2.17 ± 1.90 vs 0.98 ± 1.15; p < .0001); 93.1% of patients with inhibitors experienced joint bleeds during the 12 months compared to 55.2% of patients without inhibitors (*p* < .0001). Chronic hemophilia-related pain was more prevalent and significantly worse in the cohort with inhibitors (Fig. 1). Moderate or severe chronic pain was reported in more than half (53.4%) of patients with inhibitors and in about one-third (32.8%) of patients without inhibitors (*p* = .0105). Outcomes in the unmatched cohorts, including bleeding events, are shown in Additional file 1.

**Table 3:** Frequency of bleeds in PS-matched patients with and without inhibitors in the CHESS study^a^

Outcomes	Patients who never developed inhibitors	Patients with current inhibitors	*p*-value^b^
	*N* = 58	*N* = 58	
Bleeds in the past 12 months (major and minor bleeds)			
Mean ± SD	3.72 ± 3.95	8.29 ± 9.18	<.0001
Median (range)	3 (0.00–18.00)	6 (1.00–60.00)	
Major bleeds, *N* (%)			
Yes	22 (37.9)	47 (81.0)	<.0001
No	36 (62.1)	11 (19.0)	
Minor bleeds, *N* (%)			
Yes	48 (82.8)	58 (100.0)	.0047
No	10 (17.2)	0 (0.0)	
Joint bleeds in past 12 months			
Mean ± SD	0.98 ± 1.15	2.17 ± 1.90	<.0001
Median (range)	1 (0.00–4.00)	2 (0.00–8.00)	
Yes, *N* (%)	32 (55.2)	54 (93.1)	<.0001
No, *N* (%)	26 (44.8)	4 (6.9)	

*BMI* body mass index, *CHESS* Cost of Haemophilia across Europe – a Socioeconomic Survey, *PS* propensity score, *SD* standard deviation

^a^Patients with current inhibitors were matched to patients who had never developed an inhibitor based on demographics (age, BMI, race) and comorbidity status using propensity scores stratified by hemophilia type. Matching was performed using a preset caliper size of 0.035 to maintain the maximum sample size using the smallest caliper width

^b^*P*-values were derived from a paired t-test or Wilcoxon signed rank test for continuous post-match variables and the McNemar’s test or exact McNemar’s test for categorical variables; *p* < .05 indicates statistical significance. The McNemar’s test was not conducted for minor bleeds due to occurrence of event in 100% of inhibitor cohort

### Health resource utilization

**Fig. 2:**
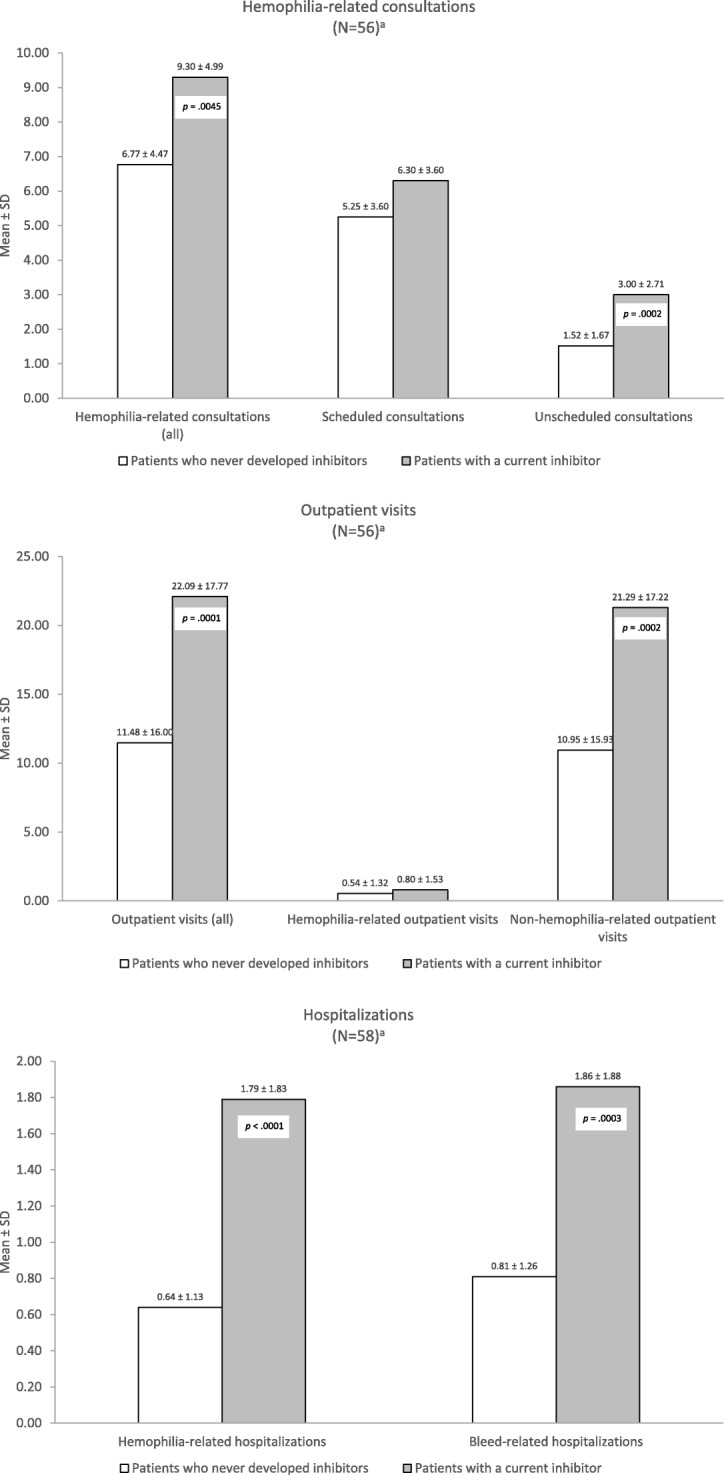
HRU frequency over 12 months for PS-matched patients with and without inhibitors in CHESS study^a^. ^a^‘N’ pertains to number of patients in each cohort after PS-matching. Patients with current inhibitors were matched to patients who had never developed an inhibitor based on demographics (age, BMI, race) and comorbidity status using propensity scores stratified by hemophilia type. Matching was performed using a preset caliper size of 0.035 to maintain the maximum sample size using the smallest caliper width. ^b^*P*-values were derived from a paired t-test or Wilcoxon signed rank test for continuous post-match variables; *p* < .05 indicates statistical significance. Abbreviations: BMI, body mass index; CHESS, Cost of Haemophilia across Europe – a Socioeconomic Survey; HRU, health resource utilization; PS, propensity score; SD, standard deviation

Fifty-six matched pairs contributed data to the analysis of HRU. Over 12 months, patients with inhibitors had consulted with hemophilia specialists significantly more often than patients without inhibitors (mean, 9.30 ± 4.99 vs 6.77 ± 4.47 visits; *p* = .0045). In addition, patients with inhibitors had significantly higher outpatient visits (mean, 22.09 ± 17.77 vs 11.48 ± 16.00; *p* = .0001), hemophilia-related (1.79 ± 1.83 vs 0.64 ± 1.13; *p* < .0001) and bleed-related admissions (1.86 ± 1.88 vs 0.81 ± 1.26; *p* = .0003) compared to their peers without inhibitors (Fig. 2).

### Physician satisfaction

Among the physicians of patients without inhibitors, 39.7% (23/58) were currently dissatisfied with their patient’s prognosis, of whom, 65.2% (15/23) believed that an optimal prognosis had been attained, and 34.8% (8/23) opined that better outcomes could be achieved. In contrast, 43.1% (25/58) of physicians of patients with inhibitors were dissatisfied with the patient’s prognosis (Table 4).

**Table 4:** Physician-reported satisfaction regarding PS-matched patients with and without inhibitors in the CHESS study^a^

Outcomes	Patients who never developed inhibitors	Patients with current inhibitors	*p*-value^b^
	*N* = 58	*N* = 58	
Physician responses to question, “Which of the following best describes your current satisfaction with the prognosis for this haemophilia patient?” *N* (%)			
*N*	58	58	
Satisfied	35 (60.3)	33 (56.9)	
Not satisfied, but I believe this is the best that can be realistically achieved for this patient	15 (25.9)	14 (24.1)	
Not satisfied, and I believe better outcomes can be achieved for this patient	8 (13.8)	11 (19.0)	
Satisfied	35 (60.3)	33 (56.9)	.6831
Not satisfied	23 (39.7)	25 (43.1)	

*Abbreviations*: *BMI* body mass index, *CHESS* Cost of Haemophilia across Europe – a Socioeconomic Survey, *PS* propensity score

^a^Patients with current inhibitors were matched to patients who had never developed an inhibitor based on demographics (age, BMI, race) and comorbidity status using propensity scores stratified by hemophilia type. Matching was performed using a preset caliper size of 0.035 to maintain the maximum sample size using the smallest caliper width

^b^*P*-values were derived from a paired t-test or Wilcoxon signed rank test for continuous post-match variables, and the McNemar’s test or exact McNemar’s test for categorical variables; *p* < .05 indicates statistical significance

## Discussion

Over the past decade, prophylactic factor replacement therapy has emerged as the standard of care for patients with severe hemophilia in developed countries where access to factor concentrates is unimpeded [28]. Still, the development of an inhibitor remains universally, the most serious treatment complication that poses a significant barrier to successful care [15, 29–32]. Assessing the burden of inhibitors in a contemporary population with severe hemophilia may therefore provide insight into the effectiveness of current treatment approaches, raise awareness of unmet needs, and assist in improving patient care.

Real-world patient-level data from the CHESS study afforded the opportunity to quantify the clinical burden of inhibitors in a cross-section of 1285 patients representing approximately 15% of the population with severe hemophilia in the EU5 [27]. The prevalence of current inhibitors was 4.5%, which approached published estimates (5–7%) for the hemophilia population, but was lower than that for patients with severe hemophilia (12–13%) [33]. Of the 1285 patients, 136 (10.6%) patients had inhibitors in the past and did not currently present with inhibitors. Considering that 84.9% of the CHESS sample had never developed inhibitors, the cumulative percentage with current and past inhibitors was 15.1%, which more closely approximated the cited range.

Consistent with other literature [34, 35], comorbidities were prevalent in nearly half (49%; 563/1149) of the total unmatched sample of all patients with hemophilia. Notably, the frequency of hypertension, which has been associated with intracranial hemorrhage and atrial fibrillation in patients with hemophilia [36, 37], was significantly higher in the inhibitor cohort (22% vs 11.5%; *p* = .0134). This may have been due to the more advanced age of the patients with inhibitors (mean, 41.9 vs 35.2 years) as age-related conditions manifest over time.

In the PS-matching analysis, patients with inhibitors were considerably more burdened compared to patients without inhibitors as evidenced by a more than two-fold increase in both the overall mean ABR and mean AJBR. Over 12 months, the vast majority of patients with inhibitors had experienced major bleeds (81%) and joint bleeds (93%). Compared to patients without inhibitors, patients with inhibitors required not only significantly more hemophilia- and bleed-related hospitalizations, but more unscheduled visits with hemophilia specialists and non-hemophilia-related outpatient visits. More than half (53.5%) experienced chronic pain interference necessitating analgesic medication. The significantly higher bleed rates in this cohort may have reflected the standard of care in the inhibitor population which may have been primarily focused on the on-demand or acute management of bleeds. Although this remains to be confirmed by examining treatment patterns in the CHESS study, the finding that more than one-third (39.7%) of physicians of inhibitor patients were dissatisfied with the patient’s prognosis may also attest to the particular challenge of managing adult patients with inhibitors.

The incremental clinical burden of inhibitors observed in the CHESS cohort mirrored the trends observed in the European Study on Orthopaedic Status of Haemophilia Patients (ESOS), a cross-sectional, case-control study of patients with hemophilia enrolled during the period from March 2004 to December 2005. In the ESOS, patients aged 14–35 years who had severe hemophilia with inhibitors (*N* = 38) had significantly worse joint pain (*p* < .05), more mobility problems (*p* < .001), and poorer orthopedic scores (p < .05) than patients without inhibitors (*N* = 49) [15]. Greater proportions of patients with inhibitors in the ESOS, irrespective of age, were also hospitalized for musculoskeletal bleeding or orthopedic procedures (16% of patients aged 14–35 years and 27% of patients aged 36–65 years [*N* = 41]) compared to patients without inhibitors (4%). The mean AJBR was comparable between the inhibitor cohorts and controls, although patients with inhibitors had significantly worse orthopedic scores [15]. Although orthopedic status was not analyzed in the CHESS sample, the higher AJBR in the inhibitor cohort (vs non-inhibitor cohort) may nonetheless signify increased morbidity as chronic joint bleeds have been associated with a greater frequency of orthopedic complications [15]. Comparing orthopedic status and the associated HRU between the PS-matched CHESS samples in future research may more precisely quantify the burden of inhibitors in the contemporary hemophilia population.

Study limitations are noted. First, as in all questionnaire-based research, the validity of the survey responses was subject to the respondents’ interpretation, recall, and accuracy in recording information. However, this limitation pertains primarily to the patients’ responses as the physicians provided data obtained from their retrospective chart reviews. Second, although we adjusted for baseline covariates, unmeasured confounding factors may have accounted for some differences in the outcomes between the PS-matched cohorts. However, the increased clinical burden of disease in the inhibitor cohort was consistent with observations in prior studies [15, 38, 39]. Third, due to the cross-sectional study design, the prevalence of current inhibitors represented a ‘point-in-time’ estimate which may have overestimated or underestimated the true burden of inhibitors. Further, we did not compare orthopedic status, which may have further differentiated the cohorts; however, a future analysis including this endpoint may be considered. Finally, although data were collected across the EU5, we did not conduct country-specific analyses due to the small sample size.

## Conclusions

Patients with inhibitors in the CHESS study exhibited greater clinical burden and utilized significantly more health resources compared to their peers without inhibitors. Physician dissatisfaction with their patients’ prognosis underscores the need for improving the standard of care for patients with inhibitors. Strategies for individualizing and improving care may also reduce the clinical burden of disease in this population.

## Additional file

Additional file 1: Frequency of bleeds, physician-reported chronic hemophilia pain by severity, HRU frequency over 12 months, and physician-reported satisfaction for patients with severe hemophilia in a CHESS study sample of unmatched cohorts with and without inhibitors. (DOCX 36 kb)

## Abbreviations

ABR: Annualized bleed rate
ADL: Activities of daily living
AJBR: Annualized joint bleed rate
BAP: Bypassing agents prophylaxis
BURQOL-RD: Social Economic Burden and Health-Related Quality of Life of Patients with Rare Diseases
CHESS: Cost of Haemophilia across Europe – a Socioeconomic Survey
ESOS: European Study on Orthopaedic Status of Haemophilia Patients
FIX: Factor IX
FVIII: Factor VIII
HRQoL: Health-related quality of life
HRU: Health resource utilization
PS: Propensity score
SD: Standard deviation
