# Supplementary material for: Inhibitor clinical burden of disease: a comparative analysis of the CHESS data
Source: Orphanet J Rare Dis. 2018 Nov 9;13:198. doi: 10.1186/s13023-018-0929-9 (PMC6230298; doi:10.1186/s13023-018-0929-9)
Supplement: Supplementary file 1 — Frequency of bleeds, physician-reported chronic hemophilia pain by severity, HRU frequency over 12 months, and physician-reported satisfaction for patients with severe hemophilia in a CHESS study sample of unmatched cohorts with and without inhibitors. (DOCX 36 kb) [file 13023_2018_929_MOESM1_ESM.docx]

Additional file 1. Frequency of bleeds, physician-reported chronic hemophilia pain by severity, HRU frequency over 12 months, and physician-reported satisfaction for patients with severe hemophilia in a CHESS study sample of unmatched cohorts with and without inhibitors

| Outcomes | All patients | Patients who never developed inhibitors | Patients with current inhibitors | *p*-value^a^ |
| --- | --- | --- | --- | --- |
|  | N=1149 | N=1091 | N=58 |  |
| *Frequency of bleeds* |  |  |  |  |
| Bleeds in the past 12 months (major and minor bleeds) |  |  |  |  |
| Mean ± SD | 4.07 ± 5.74 | 3.85 ± 5.41 | 8.29 ± 9.18 | <.0001 |
| Median (range) | 2 (0.00 – 100.00) | 2 (0.00 – 100.00) | 6 (1.00 – 60.00) |  |
| Major bleeds, N (%) |  |  |  |  |
| Yes | 486 (42.3) | 439 (40.2) | 47 (81.0) | <.0001 |
| No | 663 (57.7) | 652 (59.8) | 11 (19.0) |  |
| Minor bleeds, N (%) |  |  |  |  |
| Yes | 935 (81.4) | 877 (80.4) | 58 (100.0) | .0002 |
| No | 214 (18.6) | 214 (19.6) | 0 (0.0) |  |
| Joint bleeds in past 12 months |  |  |  |  |
| Mean ± SD | 1.04 ± 1.31 | 0.98 ± 1.25 | 2.17 ± 1.90 | <.0001 |
| Median (range) | 1 (0.00 - 9.00) | 1 (0.00 - 9.00) | 2 (0.00 – 8.00) |  |
| Yes, N (%) | 652 (56.7) | 598 (54.8) | 54 (93.1) | <.0001 |
| No, N (%) | 497 (43.3) | 493 (45.2) | 4 (6.9) |  |
| *Current hemophilia-related chronic pain* |  |  |  |  |
| Moderate to severe pain, N (%) |  |  |  |  |
| Yes | 289 (25.2) | 258 (23.6) | 31 (53.4) | <.0001 |
| No | 860 (74.8) | 833 (76.4) | 27 (46.6) |  |
| Severe pain, N (%) | 38 (3.3) | 27 (2.5) | 11 (19.0) |  |
| Moderate pain, N (%) | 251 (21.8) | 231 (21.2) | 20 (34.5) |  |
| Mild pain, N (%) | 422 (36.7) | 402 (36.8) | 20 (34.5) |  |
| No pain, N (%) | 438 (38.1) | 431 (39.5) | 7 (12.1) |  |
| *HRU frequency over 12 months* |  |  |  |  |
| Hemophilia-related consultations, mean ± SD,  median (range, min-max) |  |  |  |  |
| All hemophilia-related consultations | 6.64 ± 5.52  5 (0.00 – 80.00) | 6.50 ± 5.51  5 (0.00 – 80.00) | 9.05 ± 5.09  9 (0.00 – 25.00) | <.0001 |
| Scheduled hemophilia-related consultations | 5.12 ± 4.19  4 (0.00 – 40.00) | 5.06 ± 4.22  4 (0.00 – 40.00) | 6.14 ± 3.65  5 (0.00 – 20.00) | .0008 |
| Unscheduled hemophilia-related consultations | 1.52 ± 2.68  1 (0.00 – 60.00) | 1.44 ± 2.65  1 (0.00 – 60.00) | 2.91 ± 2.70  2 (0.00 – 10.00) | <.0001 |
| Outpatient visits |  |  |  |  |
| All outpatient visits | 9.16 ± 14.34  4 (0.00 – 171.00) | 8.48 ± 13.81  4 (0.00 – 171.00) | 21.40 ± 17.85  18 (0.00 – 63.00) | <.0001 |
| Hemophilia-related outpatient visits | 0.45 ± 1.05  0 (0.00 – 8.00) | 0.43 ± 1.02  0 (0.00 – 8.00) | 0.78 ± 1.51  0 (0.00 – 7.00) | .0803 |
| Non-hemophilia-related outpatients visits | 8.71 ± 14.17  4 (0.00 – 171.00) | 8.05 ± 13.69  3 (0.00 – 171.00) | 20.62 ± 17.29  17.5 (0.00 – 63.00) | <.0001 |
| Hospitalizations, mean ± SD, median (range, min-max) |  |  |  |  |
| Hemophilia-related hospitalizations | 0.70 ± 1.42  0 (0.00 – 15.00) | 0.63 ± 1.36  0 (0.00 – 15.00) | 1.86 ± 1.88  0 (0.00 – 8.00) | <.0001 |
| Bleed-related hospitalizations | 0.58 ± 1.29  0 (0.00 – 20.00) | 0.52 ± 1.22  0 (0.00 – 20.00) | 1.79 ± 1.83  1 (0.00 – 10.00) | <.0001 |
| *Physician-reported satisfaction* |  |  |  |  |
| Physician responses to question, ‘Which of the following best describes your current satisfaction with the prognosis for this hemophilia patient?’ N (%) |  |  |  |  |
| Satisfied | 839 (73.0) | 806 (73.9) | 33 (56.9) | .0002 |
| Not satisfied | 310 (27.0) | 285 (26.1) | 25 (43.1) |  |

^a^*P*-values were derived from a paired t-test or Wilcoxon signed rank test for continuous post-match variables and the McNemar’s test or exact McNemar’s test for categorical variables; *p* <.05 indicates statistical significance. The McNemar’s test was not conducted for minor bleeds due to occurrence of event in 100% of inhibitor cohort.

Abbreviations: CHESS, Cost of Haemophilia across Europe – an Socioeconomic Survey; HRU, health resource utilization; SD, standard deviation.
